# Where Virtual Care Was Already a Reality: Experiences of a Nationwide Telehealth Service Provider During the COVID-19 Pandemic

**DOI:** 10.2196/22727

**Published:** 2020-12-15

**Authors:** Lori Uscher-Pines, James Thompson, Prentiss Taylor, Kristin Dean, Tony Yuan, Ian Tong, Ateev Mehrotra

**Affiliations:** 1 RAND Corporation Health Care Division Arlington, VA United States; 2 Doctor on Demand San Francisco, CA United States; 3 Harvard Medical School Boston, MA United States

**Keywords:** telehealth, telemedicine, COVID-19, pandemic, infectious disease, virus, United States

## Abstract

**Background:**

The COVID-19 pandemic has led to an increase in the use of and demand for telehealth services.

**Objective:**

Here, we describe the utilization of telehealth services provided by Doctor On Demand, Inc., a well-known telehealth company in the United States, before and during the COVID-19 pandemic. We also explore how the number of virtual visits, reasons for visits, and patients served changed over time.

**Methods:**

We reported data as a percentage change from the baseline week during 2 distinct time periods: February-June 2019 and February-June 2020 based on 4 categories of visits: respiratory illness, unscheduled behavioral health, scheduled behavioral health, and chronic illness.

**Results:**

In 2020, the total visit volume increased considerably from March through April 7, 2020 (59% above the baseline) and then declined through the week of June 2 (15% above the baseline). Visits for respiratory illnesses increased through the week of March 24 (30% above the baseline) and then steadily declined through the week of June 2 (65% below the baseline). Higher relative increases were observed for unscheduled behavioral health and chronic illness visits through April (109% and 131% above the baseline, respectively) before a decline through the week of June 2 (69% and 37% above the baseline, respectively). Increases in visit volume among rural residents were slightly higher than those among urban residents (peak at 64% vs 58% above the baseline, respectively).

**Conclusions:**

Although this telehealth service provider observed a substantial increase in the volume of visits during the COVID-19 pandemic, it is interesting to note that this growth was not fueled by COVID-19 concerns but by visits for behavioral health and chronic illness. Telehealth services may play a role as a “safety valve” for patients who have difficulty accessing care during a public health emergency.

## Introduction

In response to the COVID-19 pandemic, health care delivery in the United States has changed dramatically since March 2020. As a result of stay-at-home orders, many physicians who had limited prior experience with telehealth started offering telehealth visits to support social distancing, conserve the use of personal protective equipment, and safeguard vulnerable patients from exposure to COVID-19. This transformation in health care delivery was unprecedented, and evidence about clinicians’ experiences transitioning to and maintaining telehealth services during the pandemic is still emerging.

However, when COVID-19 outbreaks emerged, many organizations were already conducting telehealth visits in high volumes. Prior to 2020, large telehealth service providers were providing millions of telehealth visits per year, offering patients immediate access to clinicians via videoconferencing visits from personal electronic devices [1].

To date, research on telehealth implementation during the COVID-19 pandemic has focused on the experiences of professionals new to telehealth [2]. Little is known about the impact of COVID-19 among telehealth services that were functional prior to the pandemic. To address this gap in the literature, we collaborated with a prominent telehealth service provider, Doctor On Demand, Inc., to describe their experience during the ongoing pandemic and to explore how the number of telehealth visits, reasons for visits, and patients served have changed over time.

## Methods

### Services Offered

Doctor On Demand is a telehealth company that provides services across all 50 US states. It delivers urgent care, behavioral health, preventive care, and chronic care services directly to consumers through its affiliations with self-insured employers and health plans. In March 2020, the company observed an increase in requests for visits and pursued several strategies to increase its capacity. They launched an online COVID-19 assessment tool and information center for mobile devices. The assessment tool, which was developed in collaboration with the Centers for Disease Control and Prevention, allowed patients to compare their symptoms to those suggestive of COVID-19 and provided recommendations regarding whether they need to seek care. The company also hired additional providers and increased the working hours of existing providers. Visits during the study period were delivered by board-certified primary care physicians, board-certified psychiatrists, and doctoral-level psychologists.

### Data Analysis

The company generated data in an aggregate form, as a percentage change from the baseline over 2 distinct time periods: February-June 2019 and February-June 2020. The baseline week was defined as February 25 to March 3 for 2019 and February 24 to March 1 for 2020. We selected this specific week-long period because it represented the tail end of the influenza season and, in 2020, it occurred before significant community transmission of COVID-19 was reported in the United States.

We plotted weekly changes in the visit volume from the baseline for all virtual visits and 4 specific categories of visits: respiratory illness (including acute respiratory infection, influenza-like illness, and potential COVID-19), unscheduled behavioral health services offered within the urgent care service staffed by primary care providers, scheduled behavioral health services (including therapy and psychiatry) offered within the behavioral health service staffed by specialty behavioral health providers, and chronic illness.

In the respiratory illness category, we combined acute respiratory infection, influenza-like illness, and suspected COVID-19 given the similarities in their presentation with fever and potential cough. Respiratory illness visits included visits for the following diagnoses: coronavirus infection; viral infection, unspecified; SARS-associated coronavirus; other coronavirus; acute nasopharyngitis; acute maxillary sinusitis; acute frontal sinusitis; acute pansinusitis; acute sinusitis; acute recurrent sinusitis; streptococcal pharyngitis; acute pharyngitis; acute tonsillitis; acute laryngitis; acute obstructive laryngitis (croup); acute upper respiratory infection, unspecified; influenza; viral pneumonia, unspecified; pneumonia; acute bronchitis; acute bronchiolitis; acute lower respiratory infection, unspecified; allergic rhinitis; chronic rhinitis; bronchitis; chronic bronchitis, unspecified; asthma, unspecified; acute bronchospasm; cough; dyspnea; shortness of breath; wheezing; nasal congestion; other disturbances of smell or taste; fever, unspecified; headache; other fatigue; and COVID-19 acute respiratory disease.

Chronic illness visits included visits for the following diagnoses: asthma, low back pain, hypertension, thyroid disorders, obesity, hypercholesterolemia/hyperlipidemia, chronic obstructive pulmonary disease, type 2 diabetes, complex diabetes, osteoarthritis, iron deficiency anemia, rheumatoid arthritis, fibromyalgia, prediabetes, lupus, ulcerative colitis, heart disease, Crohn disease, HIV, cancer, emphysema, sleep apnea, glaucoma, hemophilia, falls, hepatitis C, chronic kidney disease, end-stage liver disease, scleroderma, stroke, severe chronic kidney disease, hepatitis B, hemochromatosis, macrocytic anemia, macrocytosis, polymyalgia rheumatica, hepatitis A, joint replacement, and interstitial lung disease.

## Results

### Visit Volume

**Figure 1:**
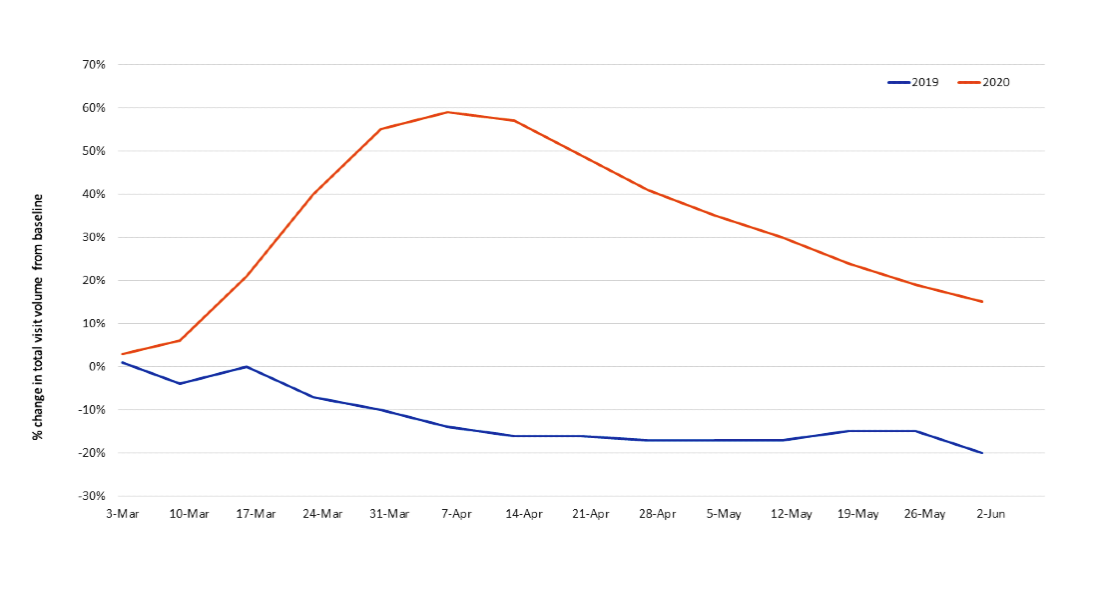
Percentage change in the total volume of virtual visits from the baseline week in 2019 and 2020. Baseline weeks: February 25 to March 3, 2019, and February 24 to March 1, 2020.

Compared to the baseline week, in 2019, total visit volume declined from March through June 2019 (Figure 1). In contrast, in 2020, total visit volume increased sharply from March to April 7, 2020 (59% above the baseline) and then steadily declined through the week of June 2, 2020 (15% above the baseline).

In 2020, during the baseline week starting February 24, respiratory illness visits represented 45% of the total visit volume, whereas the total number of visits for behavioral health (scheduled and unscheduled) and chronic illness comprised 20% and 5% of the total visit volume, respectively.

**Figure 2:**
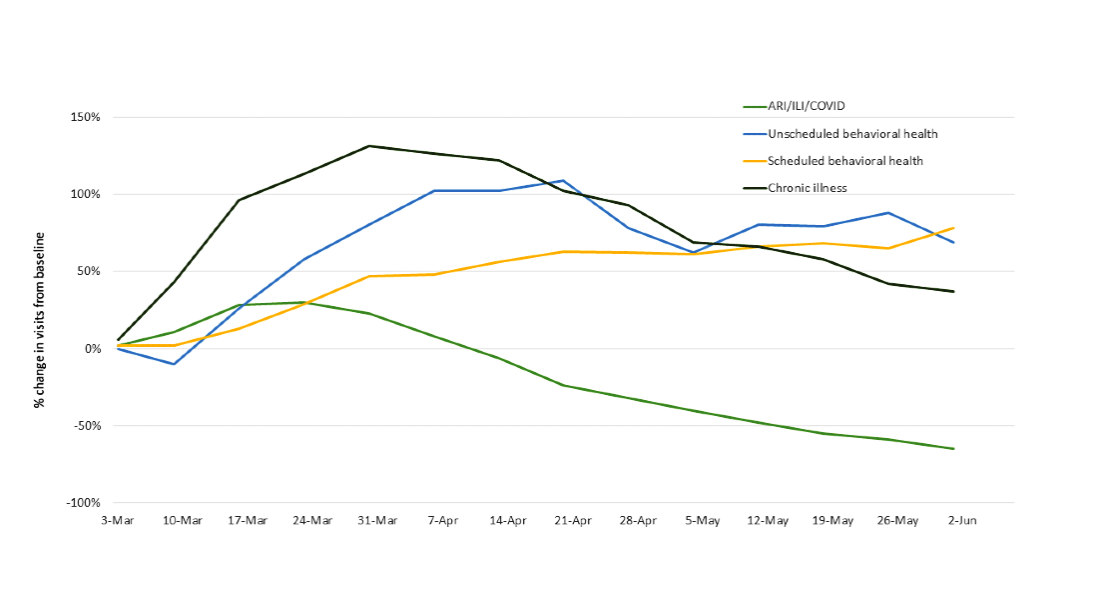
Percentage change in the total volume of virtual visits by type of visit from the baseline week in 2020. ARI: acute respiratory infection, ILI: influenza-like illness.

Moreover, in 2020, visits for respiratory illnesses modestly increased initially (peak at 30% above the baseline in the week of March 24) and then steadily declined (65% below the baseline in the week of June 2; Figure 2). In contrast, unscheduled behavioral health and chronic illness visits increased across this period, peaking at 109% in the week of April 21 and at 131% in the week of March 31, before declining to 69% and 37%, respectively—levels that were still above the baseline. In the week of June 2, 2020, respiratory illness visits represented 14% of the total visit volume, whereas behavioral health (scheduled and unscheduled) and chronic illness visits comprised 31% and 5%, respectively.

### Patients Served

In 2020, differences in visit trends by patient location were observed. All visits among urban residents peaked at 58% above baseline, whereas visits among rural residents peaked at 64% above baseline.

Individuals residing in low-income regions (mean per capita income of <US $20,000) accounted for 47% of all visits in January and February 2020 and 50% of all visits in April 2020. The proportion of patients new to the telehealth platform increased from 40% in February 2020 to 53% in April 2020.

## Discussion

Consistent with health care providers who were not focused on telehealth prior to the COVID-19 pandemic [2], the telehealth provider Doctor On Demand experienced a substantial increase in the total visit volume during the ongoing pandemic. It is noteworthy that this growth is not attributed to COVID-19 concerns; instead, behavioral health and chronic illness visits seemed to have contributed to the growth.

Although the overall growth of 59% above the baseline is substantial and similar to that reported by other telehealth services [3], it is significantly lower than the telehealth growth rate reported among in-person providers during the pandemic. For In-person providers, telehealth grew from <1% of visits to 14%-43% of visits, which corresponds to growth of >1000% [2,4]. The differences in relative growth likely suggest that the use of telehealth services among in-person providers was very low at baseline.

The overall number of telehealth visits on the Doctor On Demand platform peaked at approximately the same time as emergency department visits in the USA were at their lowest point [5]. This finding suggests that the demand for telehealth in April 2020 may have been driven in part by patient hesitation to seek in-person care. Increasing comfort with in-person care may result in a reduced demand for telehealth services as the pandemic progresses. Studies have also found that the use of telehealth services has reduced as in-person visits have rebounded [6]. However, the exposure to telehealth in the spring of 2020 may result in an increased use of these services over the long-term.

It is unclear whether the increased demand for behavioral health visits during the study period was driven by a higher incidence of mental health concerns due to pandemic-related stressors (eg, increased isolation and financial hardship) or was attributed to the reduced capacity of health care providers practicing in the community. Telehealth services may serve as a “safety valve” for patients, addressing gaps in access to the traditional (in-person) health care delivery system. Previous studies have highlighted the important role that telehealth plays during a pandemic, helping protect patients and clinicians from exposure to disease and maintaining continuity of care [7,8]. National telehealth services are especially well positioned to respond to local or regional emergencies given they have large panels of providers who are spread out geographically, and as such they can support load balancing across their networks [7]. However, in a nation affected by a pandemic, the capacity of these telehealth services is somewhat constrained by the fact that they need to hire and train additional providers to meet unexpected surges in the demand for health care. To increase the preparedness of telehealth services to scale up quickly in a national emergency, it may be necessary to maintain “reserve” providers who can be activated on a short notice.

There has been substantial discussion about the “digital divide” in health care and concerns that patients from lower-income communities may not have the necessary technology or digital literacy to participate in video-based visits. Nevertheless, it is reassuring that the proportion of telehealth visits by patients from lower-income communities was stable during the early weeks of the COVID-19 pandemic.

### Conclusions

In summary, we observed that the substantial growth in the use of telehealth services of Doctor On Demand, during the pandemic was surprisingly driven by the increase in visits for behavioral health and chronic illness. Future studies should continue to describe changes in the volume of telehealth visits and reasons for their use as the current COVID-19 pandemic progresses.
